# Halophilic Archaea Mediate the Formation of Proto-Dolomite in Solutions With Various Sulfate Concentrations and Salinities

**DOI:** 10.3389/fmicb.2019.00480

**Published:** 2019-03-12

**Authors:** Xuan Qiu, Yancheng Yao, Hongmei Wang, Anjiang Shen, Jie Zhang

**Affiliations:** ^1^State Key Laboratory of Biogeology and Environmental Geology, China University of Geosciences, Wuhan, China; ^2^Key Laboratory of Carbonate Reservoir, China National Petroleum Corporation, Hangzhou, China

**Keywords:** dolomite, halophile, sulfate, salinity, mineralization

## Abstract

In the past several decades, sulfate concentration and salinity have been considered to be the two essential hydrochemical factors in the formation of dolomite, yet arguments against this hypothesis have existed simultaneously. To clarify the effects of sulfate concentration and salinity in the mineralization of dolomite, we conducted experiments on dolomite precipitation mediated by a halophilic archaeon, *Natrinema* sp. J7-1 with various sulfate concentrations and salinities. This strain was cultured in a series of modified growth media (MGM) with salinities of 140, 200, and 280‰. Cells in the post-log phase were harvested and used to mediate the formation of dolomite in solutions with various sulfate concentrations of 0, 3, 29.8, and 100 mM and salinities of 140, 200, and 280‰. X-ray diffraction (XRD) spectra showed that proto-dolomite, monohydrocalcite, and aragonite formed in samples with cells, yet only aragonite was detected in samples without cells. Proto-dolomite was found in all biotic samples, regardless of the variation in salinity and sulfate concentration. Moreover, the relative abundances of proto-dolomite in the precipitates were positively correlated with the salinities of the media but were uncorrelated with the sulfate concentrations of the solutions. Scanning electronic microscopy (SEM) and energy dispersive spectroscopy (EDS) results showed that all the proto-dolomites were sphere or sphere aggregates with a mole ratio of Mg/Ca close to 1.0. No obvious variations in morphology and Mg/Ca were found among samples with various sulfate concentrations or salinities. This work reveals that a variation of sulfate concentration in solution (from 0 to 100 mM) does not affect the formation of dolomite mediated by halophilic archaea, but an increase of salinity (from 140 to 280‰) enhances this process. Our results indicate that under natural conditions, an increase in salinity may be more significant than the decrease of sulfates in microbe-mediated dolomite formation.

## Introduction

Dolomite is a type of carbonate mineral that mainly consists of Mg^2+^, Ca^2+^, and ${\mathrm{CO}}_{3}^{2–}$. Due to its high abundance on Earth and well-known significance in oil and gas reservoirs (Zengler et al., 1980; Warren, 2000; Gregg et al., 2015), much attention has been paid to the formation mechanisms of dolomite and the related controlling environmental factors. However, the “dolomite enigma” still remains even after over 200 years’ study (de Dolomieu, 1791; Van Tuyl, 1916; McKenzie and Vasconcelos, 2009; Gregg et al., 2015) due to the following aspects. First, researchers failed in the synthesis of dolomite with an ordered arrangement of Mg^2+^ and Ca^2+^ in the crystal (ordered dolomite) through inorganic reactions under ambient conditions in laboratories (Land, 1998; Gregg et al., 2015). Second, the modern ocean is generally oversaturated with dolomite by one or two orders of magnitude, yet very few dolomites are deposited in modern marine sediments (modern dolomite) (McKenzie and Vasconcelos, 2009). Modern dolomites are limited to saline or hypersaline environments (McKenzie and Vasconcelos, 2009). Third, the abundance of dolomite in the ocean fluctuated dramatically from ≥ 3700 M years ago (Nutman et al., 2010) to modern times, with the highest abundance during the Precambrian and a decline during the Phanerozoic eon (Zengler et al., 1980). It remains still largely unknown how environmental factors control the deposits of dolomite during the Earth’s history.

Numerous works were devoted to this enigma during the last two centuries, and both inorganic and organic pathways have been proposed for the formation of dolomite (McKenzie and Vasconcelos, 2009). For the inorganic pathway, dolomite was speculated to form either through primary precipitation [Mg^2+^ + Ca^2+^ + 2${\mathrm{CO}}_{3}^{2–}$ → MgCa(CO_3_)_2_] or secondary replacement [Mg^2+^ + 2CaCO_3_ → MgCa(CO_3_)_2_ + Ca^2+^]. The inorganic pathway well-explains the hydrothermal dolomite formation at high temperatures (>100°C) (Gregg et al., 2015; Rodriguez-Blanco et al., 2015; Kaczmarek and Thornton, 2017), but it cannot explain dolomite formation at ambient temperatures, such as 25°C (Land, 1998). Since a massive deposit of dolomite was found formed at low temperature due to the ubiquitously well-preserved fossils and sedimentary structures in ancient dolomite rocks (Blake et al., 1982; McKenzie and Vasconcelos, 2009), there should be other pathways responsible for low temperature dolomite formation. Organic pathways were proposed to be responsible for dolomite formation at low temperature. Up to now, three microbial groups, including sulfate reducing bacteria (SRB) (Vasconcelos et al., 1995; Van Lith et al., 2002), methanogens (Roberts et al., 2004; Kenward et al., 2013), and halophiles (Sánchez-Román et al., 2009; Qiu et al., 2017), have been reported to be able to mediate the formation of dolomite at ambient temperature (25∼45°C). Moreover, microbial extracellular polymeric substances (EPSs) (Krause et al., 2012; Bontognali et al., 2014), cell wall fractions (Kenward et al., 2013) and polysaccharides (Zhang et al., 2012) have also been confirmed to be able to mediate dolomite formation at low temperature. In the mineralization process, microbes not only alter microenvironments through metabolic activities but also serve as nucleation sites via negatively charged functional groups on the cell surface or EPS (Tourney and Ngwenya, 2015).

Among the various environmental factors, sulfate has been considered as the dominant inhibitor in both inorganic and organic pathways for the formation of dolomite. Baker and Kastner reported that dolomite formed in solution without sulfate but did not form with 5 mM sulfate in hydrothermal experiments at 200°C (Baker and Kastner, 1981; Kastner, 1984). Dissolved sulfate was speculated to be tightly bound to Mg^2+^ in the form of an [Mg^2+^-${\mathrm{SO}}_{4}^{2–}$] complex, which decreased the activities of Mg^2+^ and subsequently impeded the loading of Mg^2+^ onto the crystal of dolomite. The hypothesis of sulfate inhibition on dolomite formation became widely accepted by geologists, as it well-explained the downtrend of dolomite along the Earth’s history with the increase of sulfate concentration in the ocean. The formation of dolomite was thought to be favored in the ancient ocean with low sulfate concentrations [e.g., 7–10 mM in the Neoproterozoic era (Kah et al., 2004)] and limited in the modern ocean with high sulfate concentrations (∼29 mM). Moreover, the inhibition of sulfate was also accepted by geomicrobiologists, who confirmed the roles of SRB in mediating dolomite formation (Vasconcelos et al., 1995; Vasconcelos and McKenzie, 1997). However, this hypothesis was challenged recently (Kenward et al., 2013; Wang et al., 2016). Field investigations showed that many locations with modern dolomite deposits were rich in sulfate, including the saline lakes in Western Australia (9.8–460 mM) (De Deckker and Last, 1988) and the Lagoa Vermelha lagoon in the east coast of Brazil (42–60 mM) (Vasconcelos and McKenzie, 1997). The reduction of sulfate by SRB in the above settings did not obviously decrease the concentration of sulfate. Moreover, culture experiments demonstrated that dolomite formation was successfully mediated by halophiles on agar plates with a relatively high sulfate concentration (up to 56 mM), during which the halophilic microorganisms did not reduce sulfate (Sánchez-Román et al., 2009). Further, Raman analysis in the study by Wang et al. (2016) suggested that most soluble ${\mathrm{SO}}_{4}^{2–}$ might not bind with Mg^2+^ at low temperatures (≤25°C). Nonetheless, the studies above did not thoroughly clarify the effect of sulfate on dolomite formation. In the work of Sánchez-Román et al. (2009), dolomite precipitated on semi-solid plates, which were solidified by agar, a type of polysaccharide mixture. Since similar polysaccharides had been reported to be able to mediate the formation of proto-dolomite (Zhang et al., 2012), the possibility remained agar neutralized the sulfate-dependent inhibition of dolomite formation, and therefore sulfate inhibition could not be thoroughly excluded. Besides that, the sulfate concentrations in the work of Sánchez-Román et al. (2009) referred to the values of the media before solidification. However, the activity of sulfate in the media before and after solidification might be largely different. In addition, the lowest sulfate concentration tested in the study of Wang et al. (2016) was 500 mM, which was much higher than the average sulfate concentration in the modern oceans (29 mM). The gap is even larger when compared to the sulfate concentration in ancient oceans. Therefore, many issues related to the inhibition of sulfate on dolomite formation remain un-addressed and further investigation is needed.

Since modern dolomite mainly deposited in saline or hypersaline settings (De Deckker and Last, 1988; Rosen et al., 1989; Van Lith et al., 2002; Meister et al., 2011) and microbe-mediated dolomite usually formed in solutions with moderate or high salinities in lab experiments (Vasconcelos et al., 1995; Warthmann et al., 2000; Sánchez-Román et al., 2009; Bontognali et al., 2012; Kenward et al., 2013; Qiu et al., 2017), we proposed that salinity was a critical environmental factor controlling dolomite formation and halophile was an effective microbial group for organic dolomite formation (Qiu et al., 2017). However, the combined influence of sulfate and salinity on halophile-mediated dolomite formation was still unclear. To investigate the influence of sulfate on dolomite formation under different salinities, we grew a moderate halophilic archaeon, *Natrinema* sp. J7-1, under varying salinities. Harvested cells were used to mediate dolomite formation in solutions with varying sulfate concentrations and salinities. We expect to clarify the effects of sulfate and salinity on halophile-mediated dolomite formation.

## Materials and Methods

### Strain and Medium

*Natrinema* sp. J7-1 (CCTCC AB 2012856) is a moderate halophilic archaeon isolated from a salt mine (Shen and Chen, 1994; McGenity et al., 1998) and was provided by Dr. Xiangdong Chen of Wuhan University. This strain was able to mediate the formation of proto-dolomite, as demonstrated in our previous works (Duan et al., 2017; Yao et al., 2018). *N*. sp. J7-1 was maintained in a modified growth medium (MGM) (Holmes and Dyall-Smith, 1990) with the following components: 2.46 M NaCl, 88.67 mM MgCl_2_, 85.37 mM MgSO_4_, 56.38 mM KCl, 5.05 mM CaCl_2_, 5.00 g/L peptone, and 3.00 g/L yeast extract. The initial pH value of the medium was adjusted to 7.50 with 8 M NaOH solution, and the medium was autoclaved for 30 min at 121°C.

### Growth Curve

*Natrinema* sp. J7-1 was cultured in MGM media amended to have salinities of 140, 200, and 280‰ by altering the dosage of NaCl. A 10 mL aliquot of preserved culture was inoculated into 1000 mL of fresh medium and then incubated at 45°C with shaking at 150 rpm. The optical density at 600 nm (OD_600_) was measured every 12 h by a spectrophotometer (UT-180, Beijing Puxi), and the growth curves were plotted as incubation time versus OD_600_.

### Mineralization Experiments

*Natrinema* sp. J7-1 was cultured in the same media and under the same conditions as mentioned in Section “Growth Curve.” Microbial cells in post-log phase were collected for mineralization experiments due to their high integrity and considerable biomass. The cultures reached the middles of the post log-phases after growing for 60, 48, and 60 h in media with salinities of 140, 200, and 280‰, respectively. The cells were collected by centrifugation at 9000 × *g* for 5 min, followed by washing three times with NaCl-H_2_O solutions to thoroughly remove residual media. The NaCl-H_2_O solutions were prepared by dissolving different amounts of NaCl in double distilled water (ddH_2_O, Ω > 18.25 M cm^-1^ at 25°C) to reach final salinities of 140, 200, and 280‰ and used correspondingly with the salinity of the media. The washed cells were re-suspended individually in corresponding NaCl-H_2_O solutions to reach a final OD_600_ value of 2.0. A 10.0 mL aliquot of the diluted cell suspension was transferred into a 50 mL tube (Corning, NY, United States), followed by the addition of specific amounts of Na_2_SO_4_, MgCl_2_, CaCl_2_, Na_2_CO_3_, NaCl, and ddH_2_O (Table 1). The final concentrations of Ca^2+^, Mg^2+^, and ${\mathrm{CO}}_{3}^{2–}$ were 10.6, 106, and 20 mM, respectively. The final concentrations of ${\mathrm{SO}}_{4}^{2–}$ were 0 mM, 3 mM (corresponding to the ocean during the Permian-Triassic boundary), 29.8 mM (corresponding to the modern ocean) or 100 mM (a very high value). Blank controls were made by replacing the 10 mL cell solution with a 10 mL NaCl-H_2_O solution with salinities of 140, 200, and 280‰, respectively. The Na_2_CO_3_ solution was slowly added with an aliquot of 100 μL for every single addition. Simultaneously, the tube was shaken by hand to thoroughly mix the Na_2_CO_3_ with the solution. Subsequently, the centrifuge tubes were incubated at 45°C with shaking at 150 rpm for 72 h. Precipitates were collected by centrifugation at 9000 × *g* for 5 min. The pellets were washed three times with ddH_2_O and then dried in a freeze dryer (Alpha1-2/LD, Christ, German).

**Table 1:** The components in the solutions prepared for mineralization experiments.

Salinity (‰)	Cell suspension (mL)	NaCl solution (mL)	1.111 M Na_2_SO_4_ (mL)	1.178 M MgCl_2_ (mL)	1.178 M CaCl_2_ (mL)	NaCl (g)	0.200 M Na_2_CO_3_ (mL)	ddH_2_O
140	10.000	0.000	0	2.000	0.200	2.814	2.000	Match to 20 mL
			0.060			2.804		
			0.596			2.718		
			2.000			2.490		

140	0.000	10.000	0	2.000	0.200	2.814	2.000	Match to 20 mL
(Control)			0.060			2.804		
			0.596			2.718		
			2.000			2.490		

200	10.000	0.000	0	2.000	0.200	4.148	2.000	Match to 20 mL
			0.060			4.146		
			0.596			4.054		
			2.000			3.832		

200	0.000	10.000	0	2.000	0.200	4.148	2.000	Match to 20 mL
(Control)			0.060			4.146		
			0.596			4.054		
			2.000			3.832		

280	10.000	0.000	0	2.000	0.200	5.926	2.000	Match to 20 mL
			0.060			5.924		
			0.596			5.832		
			2.000			5.610		

280	0.000	10.000	0	2.000	0.200	5.926	2.000	Match to 20 mL
(Control)			0.060			5.924		
			0.596			5.832		
			2.000			5.610		

### Characterization of the Precipitates

The mineral phases of the precipitates were detected via X-ray diffraction (XRD) (Bruker AXS D8-Focus, Germany) using Cu-Kα radiation. The peaks of the minerals were detected from 3 to 70° (2𝜃) with a step size of 0.01° and scan speed of 10°/min. The XRD spectra were analyzed using the Jade 6.5 software. The percentages of the minerals in the precipitates were calculated by the Software of Xpowder 12. The mole percentage of Mg^2+^ in dolomite was calculated based on the empirical equation of Goldsmith et al. (1961) and Zhang et al. (2010). XRD analysis was conducted at the School of Material and Chemical Engineering at the China University of Geoscience (Wuhan).

The morphology and elements of the precipitates were determined by scanning electron microscopy (SEM, FEI Quanta 450 FEG, United States) with an X-ray Energy Dispersive Spectrometer (EDS, SDD Inca X-Max 50, Britain). Dried precipitates were adhered onto SEM stubs with double-sided conductive tapes, followed by coating with Pt for image observation. The accelerating voltages of the SEM and EDS were 15 and 20 kV, respectively. SEM analysis was done at the State Key Laboratory of Geobiology and Environmental Geology at the China University of Geoscience (Wuhan).

## Results

### The Growth of *N*. sp. J7-1 Under Various Salinities

**FIGURE 1:**
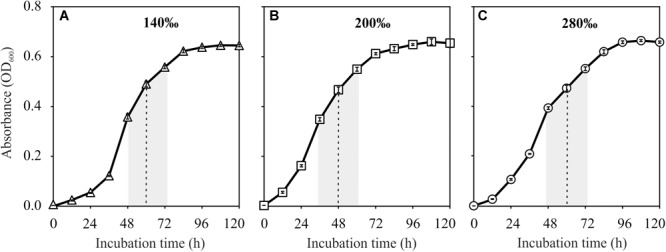
Growth curves of *Natrinema* sp. J7-1 cultured in media with salinities of 140‰ **(A)**, 200‰ **(B)**, and 280‰ **(C)**. Error bars were showed in the legends. The post-log phases and the sampling times were indicated by the shaded areas and dashed lines, respectively.

*Natrinema* sp. J7-1 grew well in media with salinities of 140, 200, and 280‰. All the cultures reached their stationary phase after approximately 96 h with the highest optical absorptions of approximately 0.65. However, the duration to the middle of post-log phase was about 48 h for the culture with a salinity of 200‰, faster than those of the other two cultures with salinities of 140 and 280‰, which were approximately 60 h (Figure 1). Based on these growth curves, the sampling times (the middle of post log-phase) were determined to be 60, 48, and 60 h for the cultures that grew in media with salinities of 140, 200, and 280‰, respectively.

### Mineral Phases of the Precipitates Induced by *N*. sp. J7-1

**FIGURE 2:**
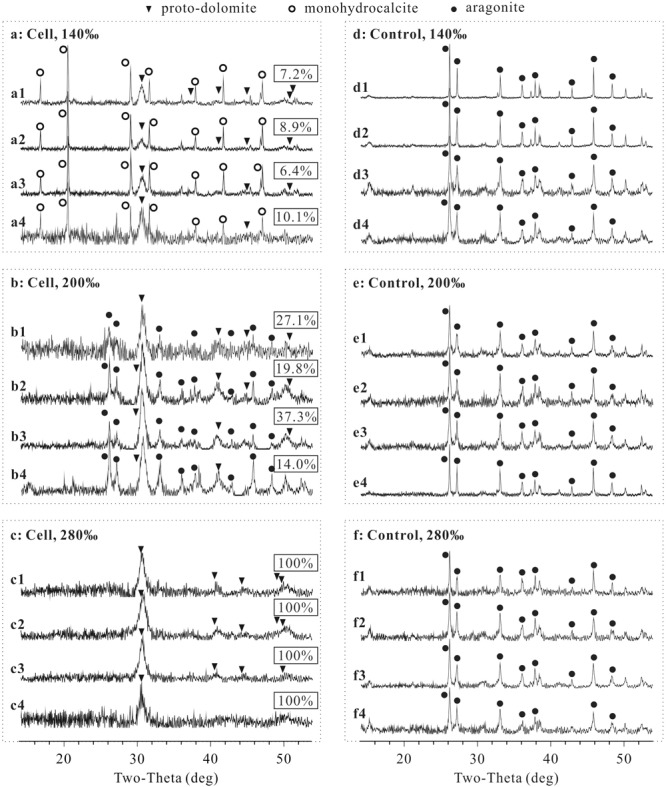
X-ray diffraction (XRD) spectra of precipitates and the percentages of proto-dolomite therein mediated by *N*. sp. J7-1 in media with various sulfate concentrations and salinities. The concentrations of sulfate in “X1,” “X2,” “X3,” and “X4” are 0, 3, 29.8, and 100 mM, respectively, “X” = a, b, c, d, e, or f. Only partial XRD spectra (2𝜃 = 14 to 54°) are presented here, as all peaks are located within this zone. The rectangular labels show the percentages of proto-dolomite in each precipitate. **(a)** is the group with cells mineralized in solution with a salinity of 140‰, **(b)** is the group with cells mineralized in solution with a salinity of 200‰, **(c)** is the group with cells mineralized in solution with a salinity of 280‰, **(d)** is the group without cells mineralized in solution with a salinity of 140‰, **(e)** is the group without cells mineralized in solution with salinity of 200‰, and **(f)** is the group without cells mineralized in solution with a salinity of 280‰.

Proto-dolomite, monohydrocalcite and aragonite formed in samples with cells of *N*. sp. J7-1 (Figure 2A–C), yet only aragonite was presented in the precipitates of the controls (Figure 2D–F). Notably, proto-dolomite but not ordered-dolomite was identified due to the lack of “ordering peaks” (101, 015, and 021) on the spectra. The variation of sulfate concentrations did not affect the features of the precipitates under all salinities tested, yet both the mineral phases and their relative abundances changed with salinities (Figure 2A1–A4,B1–B4,C1–C4). Monohydrocalcite was the major mineral and proto-dolomite was the second major mineral in the precipitates induced by cells harvested from the medium with a salinity of 140‰ (Figure 2A1–A4). With a salinity of 200‰, aragonite became the major component and proto-dolomite was still the second major mineral (Figure 2B1–B4). In the group with the highest salinity (280‰), proto-dolomite was the unique mineral in the precipitates (Figure 2C1–C4). The percentages of proto-dolomite increased from 6.4∼10.1% in the samples with a salinity of 140‰ to 14.0∼37.3% in the samples with a salinity of 200‰, and further increased to 100% in the samples with a salinity of 280‰ (Figure 2, *p* < 0.01). These results demonstrated that halophilic archaeon *N*. sp. J7-1 were able to mediate the formation of proto-dolomite. The variation of sulfate concentration from 0 to 100 mM did not affect the formation of proto-dolomite. However, the elevation of salinity increased the percentages of proto-dolomite in the precipitates mediated by cells of *N*. sp. J7-1.

The percentage of Mg^2+^ in the proto-dolomite ranged from 41.0 to 48.6%, calculated from the position of *d*(104) on the XRD spectra. Within the groups having the same salinity, no obvious relationship was found between the percentage of Mg^2+^ and the sulfate concentration. Among the three groups (salinity = 140, 200, and 280‰), the average percentage of Mg^2+^ increased weakly with salinity (Table 2), though this correlation was not statistically significant (*p* = 0.266).

**Table 2:** The calculated percentages of Mg^2+^ in proto-dolomite based on the empirical equation of Goldsmith et al. (1961) and Zhang et al. (2010).

Salinity	Sulfate concentration	*d*(104)	Mg^2+^	Average Mg^2+^
(‰)	(mM)	(Å)	(%)	(%)
140	0	2.919	41.0	42.48 ± 1.93
	3	2.914	42.5	
	29.8	2.918	41.2	
	100	2.905	45.2	

200	0	2.908	44.3	44.25 ± 1.37
	3	2.905	45.2	
	29.8	2.915	42.3	
	100	2.905	45.2	

280	0	2.905	45.2	45.95 ± 3.29
	3	2.895	48.4	
	29.8	2.894	48.6	
	100	2.917	41.6	

### Morphology and the Mole Mg^2+^ Percentage of the Minerals

**FIGURE 3:**
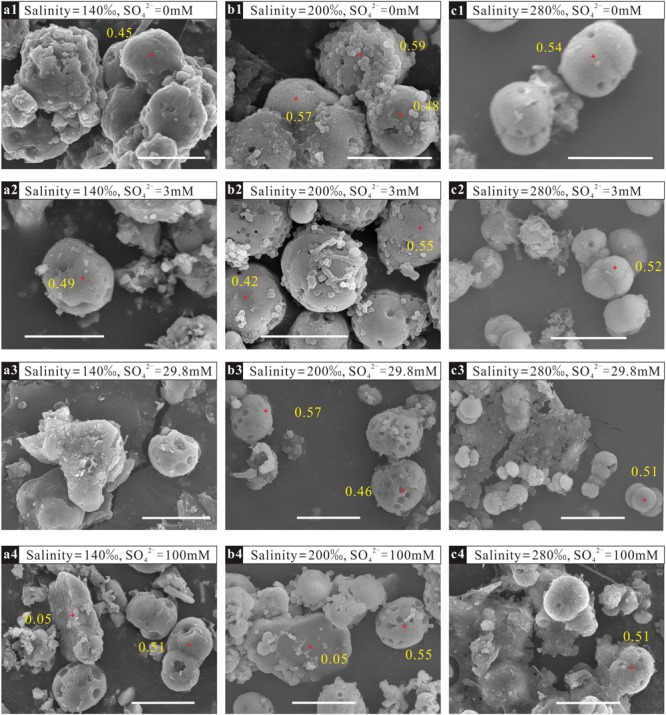
Morphologies and percentages of Mg^2+^ in precipitates mediated by *N*. sp. J7-1 in solutions with different sulfate concentrations and salinities. The bars in the figures are all 10 μm. **(a1–a4)** are samples mineralized in solutions with the same salinity of 140‰ but various sulfate concentrations of 0 mM, 3 mM, 29.8 mM, and 100 mM, respectively; **(b1–b4)** are samples mineralized in solutions with the same salinity of 200‰ but various sulfate concentrations of 0 mM,3 mM, 29.8 mM, and 100 mM, respectively; **(c1–c4)** are samples mineralized in solutions with the same salinity of 280‰ but various sulfate concentrations of 0 mM, 3 mM, 29.8 mM, and 100 mM, respectively.

The morphologies of the minerals mediated by *N*. sp. J7-1 were spherical, blocky or amorphous (Figure 3). EDS analysis demonstrated high Mg^2+^ contents (>45%, compared with Ca^2+^) on spheres with a diameter of 5–10 μm, which was the proto-dolomite. Minerals with other shapes with low Mg^2+^ contents were aragonite or monohydrocalcite.

## Discussion

### The Relationship Between “Dolomite” and “Proto-Dolomite”

In the past several decades, the formations of dolomite mediated by microbes or organic molecules have attracted much attention (Vasconcelos et al., 1995; Roberts et al., 2004; Bontognali et al., 2012; Krause et al., 2012; Kenward et al., 2013; Qiu et al., 2017). In some studies, the precipitates were identified as dolomite solely based on the approximately equal percentages of Mg^2+^ and Ca^2+^ (Warthmann et al., 2000; Bontognali et al., 2012), whereas ordered arrangement of these two cations was also considered as the second criterion in other works (Vasconcelos et al., 1995; Roberts et al., 2004; Krause et al., 2012; Kenward et al., 2013; Qiu et al., 2017). Ca-Mg-CO_3_ with equal content of Mg^2+^ and Ca^2+^ but no ordered arrangement was named “proto-dolomite” (Gaines, 1977) or “disordered dolomite” (Zhang et al., 2012) or “Very High Mg-Calcite” (Gregg et al., 2015); it was speculated that these compounds represented the precursor of ordered dolomite (Gaines, 1977; Gregg et al., 2015; Pina, 2015). This work refers to the mineral terminology according to Gaines (1977). The Ca-Mg carbonate was identified as “proto-dolomite” since there were peaks of *d*(104) indicating Mg/Ca≈1, but no peaks indicating ordered arrangement of Mg^2+^ and Ca^2+^ on their XRD spectra (Figure 2).

In this study, short period of mineralization (72 h) may be the main reason for the absence of ordered dolomite. It has been reported that the cation ordering of a Holocene dolomite increased from recent layer to ancient layer (Gregg et al., 1992), suggesting the transformation from disordered dolomite to ordered dolomite with age. However, how this alteration proceeded at ambient conditions is unclear (Land, 1998; Gregg et al., 2015). Despite of lacking ordered dolomite, we clearly demonstrated that the increase of salinity facilitated the formation of proto-dolomite mediated by *N*. sp J7-1, while the increase of sulfate concentration didn’t show such an effect. These results enhance our understanding of the effect of salinity and sulfate on microbial dolomite formation.

### Sulfate Does Not Inhibit Dolomite Formation Under Ambient Conditions

In the past several decades, sulfate was generally considered to be an inhibitor of the formation of dolomite through both inorganic and organic pathways, yet the evidence has remained contradictory. Dissolved sulfate was believed to bind tightly with Mg^2+^ in solution due to the high association constant of the [Mg^2+^-${\mathrm{SO}}_{4}^{2–}$] complex (Smith and Martell, 1976). Thus, the incorporation of Mg^2+^ into the crystal of dolomite would be impeded by the presence of dissolved sulfate, especially at high concentrations. This perspective was proven by hydrothermal experiments (Baker and Kastner, 1981; Kastner, 1984; Morrow and Ricketts, 1988), in which the addition of 5 mM sulfate inhibited the formation of dolomite from calcite at 200°C (Baker and Kastner, 1981). Similarly, Morrow and Ricketts reported that the formation of dolomite from calcite slowed down in the presence of < 4 mM dissolved sulfate and completely stopped when the concentration of sulfate reached 4 mM at high temperature (215∼225°C) (Morrow and Ricketts, 1988). At low temperatures, the impact of sulfate on inorganic dolomite formation was unclear as few attempts at low temperature dolomite formation succeeded. An increase in sulfate concentration from 0 to 5 mM decreased the attachment of Mg^2+^ onto the crystal surface of dolomite, while the inverse impact occurred when the concentration of sulfate was above 5 mM (Brady et al., 1996). This work matched well with the results of hydrothermal experiments with low sulfate concentration (<5 mM) (Baker and Kastner, 1981). The inhibition of sulfate was also widely accepted by researchers supporting organic dolomite formation, especially for those who confirmed the role of SRB. With the metabolic activities of SRB, sulfate would be removed, consequently releasing Mg^2+^ from the [Mg^2+^-${\mathrm{SO}}_{4}^{2–}$] complex and facilitating the formation of dolomite. However, halophile-mediated dolomite was found with sulfate concentrations from 0 to 56 mM on agar plates (Sánchez-Román et al., 2009), as well as in solutions with sulfate concentrations ranging from 0 to 100 mM in this study (Figure 1). The concentration of sulfate kept constant during the process of mineralization as the halophiles in these experiments could not reduce and remove sulfate from the media. These results demonstrated that sulfate did not inhibit the formation of dolomite. Raman spectra analysis of artificial MgSO_4_ solution showed that the association of Mg^2+^-${\mathrm{SO}}_{4}^{2–}$ was very weak at ambient temperature, which suggested that the Mg^2+^ in natural waters was generally unassociated with sulfate despite of the variation of sulfate concentration (Wang et al., 2016). Therefore, it was reasonable to find that sulfate concentration didn’t affect the formation of dolomite in our experiments.

To summarize, sulfate did inhibit dolomite formation in the inorganic pathway at high temperature (>200°C), but not at low temperature with microorganisms.

### High Salinity Enhances Dolomite Formation

Salinity is another environmental factor proposed to affect dolomite formation. Elevation of salinity in a natural setting is generally achieved by evaporation. This process may affect the inorganic pathways of dolomite formation in two contradictory ways. On the one hand, evaporation increases the concentration of Ca^2+^ and Mg^2+^ and consequently elevates the saturation index of the solution to dolomite (Van Lith et al., 2002), which favors the formation of dolomite. On the other hand, evaporation also disturbs the nucleation of dolomite by adding more foreign ions (Folk and Land, 1975). Some researchers proposed that a decrease in salinity favored for dolomite formation (Folk and Land, 1975; Morrow, 1978), whereas others held the reversed opinion (Van Lith et al., 2002; Gabellone and Whitaker, 2016). In contrast, the role of salinity in organic dolomite formation was more consistent, as elevation of salinity always enhanced the formation of dolomite (Voegerl, 2014; Rivadeneyra et al., 2016; Qiu et al., 2017). In this study, the percentage of proto-dolomite in the precipitates mediated by *N*. sp J7-1 increased with the elevation of the salinity in the media (*p* < 0.01) (Figure 2). It was shown that an increase in salinities increased the densities of carboxylated functional groups on the cell surfaces of *Desulfovibrio brasiliensis* (Voegerl, 2014), *Haloferax sulfurifontis* (Voegerl, 2014) and *H*. *volcanii* DS52 (Qiu et al., 2017), all of these strains were able to mediate the formation of dolomite (Vasconcelos and McKenzie, 1997; Kenward et al., 2013; Qiu et al., 2017). Carboxyl groups have been speculated to be able to mediate the equal loading of Ca^2+^ and Mg^2+^ during the nucleation process of dolomite (Kenward et al., 2013). In view of the common distribution of modern dolomite in settings with high salinities (Peterson et al., 1963; McKenzie, 1981; De Deckker and Last, 1988; Vasconcelos and McKenzie, 1997), we hypothesized that high salinity might be more important than low sulfate concentration in dolomite formation.

### The Roles of Halophile in Dolomite Formation

Modern dolomites are generally limited to hypersaline and saline environments, including sabkhas (McKenzie, 1981; Brauchli et al., 2015), lagoons (Vasconcelos and McKenzie, 1997; Van Lith et al., 2002), reefs (Mitchell et al., 1987), and saline lakes (De Deckker and Last, 1988; Rosen et al., 1989; Meister et al., 2011). Microbial mats (Brauchli et al., 2015), microbial enrichments (Vasconcelos et al., 1995), microbial pure strains (Bontognali et al., 2012), and microbial organic matter in these environments (Krause et al., 2012; Bontognali et al., 2014) were demonstrated able to mediate the formation of dolomite/proto-dolomite. Without microbes and organic additives, dolomite did not form at ambient conditions in solution with a high saturation index for as long as 32 years (Land, 1998). These studies consistently confirmed that microorganisms were critical for the deposition of dolomite in settings with high salinity.

To survive in water with high salinity, many adaptive strategies were evolved by microbes, including releasing abundant EPS to the extracellular space (Liu and Buskey, 2000; Qiu et al., 2012; Biswas and Paul, 2017), modifying the components on the cell surfaces (Guan et al., 2012; Voegerl, 2014; Qiu et al., 2017) and accumulating organic components and K^+^ in intracellular spaces (Oren, 2013). As mentioned above, abundant negative functional groups occurred on both the cell surface and EPS, which could serve as nucleation sites for Mg-CaCO_3_ due to their excellent binding capacity with cations, e.g., Mg^2+^ and Ca^2+^. In addition, negative functional groups on organic molecules decreased the dielectric constant of the solution and consequently facilitated the dehydration of the [Mg^2+^-H_2_O] complex (Zhang et al., 2012). Compared with other negative functional groups, carboxyls on the cell surface have been proposed to be essential for dolomite formations as physiochemical analysis demonstrated that the presence of carboxyls enhanced the equal loading of Mg^2+^ and Ca^2+^ onto the crystal of Mg-CaCO_3_ (Kenward et al., 2013; Qiu et al., 2017). With increasing salinity, the carboxyl content on the surface of *H*. *volcanii* DS52 increased, and consequently, proto-dolomite formed at relatively high salinities (Qiu et al., 2017). In this study, the capacity of *N*. sp. J7-1 to mediate dolomite formation was also enhanced by the elevation of salinity.

Hypersaline environments are not only inhabited by halophilic and halotolerant chemoorganotrophic archaea, but also by SRB, methanogens, et al. (Oren, 2013). Thus, it is reasonable to speculate that SRB inhabited there should also adopt those anti-salt strategies. Similar to typical halophiles, elevation of salinity may also increase the carboxyl groups on the cell surfaces of SRBs, methanogens, and other microbes in saline and hypersaline environments, which subsequently favors for the formation of dolomite (Voegerl, 2014; Qiu et al., 2017).

## Conclusion

A halophilic archaeon, *N*. sp. J-1, successfully mediated the formation of proto-dolomite under various sulfate concentrations (0, 3, 29.8, and 100 mM) and salinities (140, 200, and 280‰). Mineral phases did not change with the variation of sulfate concentrations, whereas the percentage of proto-dolomite in the precipitates increased with the increase of salinity from 140 to 280‰. This work clearly showed that elevated salinities facilitated the formation of proto-dolomite by microbes at ambient temperatures, while changing sulfate concentrations had no effect.

## Author Contributions

XQ and HW designed the experiments. XQ and YY performed the laboratory work and analyzed the data. XQ, HW, AS, and JZ wrote the manuscript. All authors approved the final manuscript.

## Conflict of Interest Statement

The authors declare that the research was conducted in the absence of any commercial or financial relationships that could be construed as a potential conflict of interest.

## References

[B1] Baker P. A. Kastner M. (1981). Constraints on the formation of sedimentary dolomite. *Science* 213 214–216. 10.1126/science.213.4504.214 17782787

[B2] Biswas J. Paul A. (2017). diversity and production of extracellular polysaccharide by halophilic microorganisms. *Biodivers. Int. J.* 1 1–9. 10.15406/bij.2017.01.00006 11938472

[B3] Blake D. F. Peacor D. R. Wilkinson B. H. (1982). The sequece and mechanism of low temperature dolomite formation calcian dolomites in a pennsylvanian enchinoderm. *J. Sediment. Petrol.* 52 59–70.

[B4] Bontognali T. R. McKenzie J. A. Warthmann R. J. Vasconcelos C. (2014). Microbially influenced formation of Mg-calcite and Ca-dolomite in the presence of exopolymeric substances produced by sulphate-reducing bacteria. *Terra Nova* 26 72–77. 10.1111/ter.12072

[B5] Bontognali T. R. R. Vasconcelos C. Warthmann R. J. Lundberg R. McKenzie J. A. (2012). Dolomite-mediating bacterium isolated from the sabkha of Abu Dhabi (UAE). *Terra Nova* 24 248–254. 10.1111/j.1365-3121.2012.01065.x

[B6] Brady P. V. Krumhansl J. L. Papenguth H. W. (1996). Surface complexation clues to dolomite growth. *Geochim. Cosmochim. Acta* 60 727–731. 10.1016/0016-7037(95)00436-X

[B7] Brauchli M. McKenzie J. A. Strohmenger C. J. Sadooni F. Vasconcelos C. Bontognali T. R. R. (2015). The importance of microbial mats for dolomite formation in the Dohat Faishakh sabkha, Qatar. *Carbonates Evaporites* 31 339–345. 10.1007/s13146-015-0275-0

[B8] De Deckker P. Last W. M. (1988). Modern dolomite deposition in continental, saline lakes, western Victoria. *Aust. Geol.* 16 29–32. 10.1130/0091-7613(1988)016<0029:MDDICS>2.3.CO;2

[B9] de Dolomieu D. (1791). Sur un genre de pierres calcaires trespeu effervescentes avec les acides et phosphorescentes parla collision. *J. Phys.* 39 3–10.

[B10] Duan Y. Yao Y. C. Qiu X. Wang H. M. (2017). Dolomite formation facilitated by three halophilic archaea. *Earth Sci.* 42 389–396.

[B11] Folk R. L. Land L. S. (1975). Mg/Ca ratio and salinity: two controls over crystallization of dolomite. *AAPG Bull.* 59 60–68.

[B12] Gabellone T. Whitaker F. (2016). Secular variations in seawater chemistry controlling dolomitization in shallow reflux systems: insights from reactive transport modelling. *Sedimentology* 63 1233–1259. 10.1111/sed.12259

[B13] Gaines A. M. (1977). Protodolomite redefined. *J. Sediment. Res.* 47 543–546. 10.1306/212F71D0-2B24-11D7-8648000102C1865D

[B14] Goldsmith J. Graf D. Heard H. (1961). Lattice constants of the calcium-magnesium carbonates. *Am. Mineralog.* 46 453–457.

[B15] Gregg J. M. Bish D. L. Kaczmarek S. E. Machel H. G. (2015). Mineralogy, nucleation and growth of dolomite in the laboratory and sedimentary environment: a review. *Sedimentology* 62 1749–1769. 10.1111/sed.12202

[B16] Gregg J. M. Howard S. A. Mazzullo S. J. (1992). Early diagenetic recrystallization of Holocene (< 3000 years old) peritidal dolomites, Ambergris Cay, Belize. *Sedimentology* 39 143–160. 10.1111/j.1365-3091.1992.tb01027.x

[B17] Guan Z. Naparstek S. Calo D. Eichler J. (2012). Protein glycosylation as an adaptive response in Archaea: growth at different salt concentrations leads to alterations in Haloferax volcanii S-layer glycoprotein N-glycosylation. *Environ. Microbiol.* 14 743–753. 10.1111/j.1462-2920.2011.02625.x 22029420 PMC3414426

[B18] Holmes M. Dyall-Smith M. (1990). A plasmid vector with a selectable marker for halophilic archaebacteria. *J. Bacteriol.* 172 756–761. 10.1128/jb.172.2.756-761.1990 2105303 PMC208503

[B19] Kaczmarek S. E. Thornton B. P. (2017). The effect of temperature on stoichiometry, cation ordering, and reaction rate in high-temperature dolomitization experiments. *Chem. Geol.* 468 32–41. 10.1016/j.chemgeo.2017.08.004

[B20] Kah L. C. Lyons T. W. Frank T. D. (2004). Low marine sulphate and protracted oxygenation of the proterozoic biosphere. *Nature* 431 834–838. 10.1038/nature02974 15483609

[B21] Kastner M. (1984). Control on dolomite formation. *Nature* 311 410–411. 10.1038/311410b06207433

[B22] Kenward P. A. Fowle D. A. Goldstein R. H. Ueshima M. González L. A. Roberts J. A. (2013). Ordered low-temperature dolomite mediated by carboxyl-group density of microbial cell walls. *AAPG Bull.* 97 2113–2125. 10.1306/05171312168

[B23] Krause S. Liebetrau V. Gorb S. Sanchez-Roman M. McKenzie J. A. Treude T. (2012). Microbial nucleation of Mg-rich dolomite in exopolymeric substances under anoxic modern seawater salinity: new insight into an old enigma. *Geology* 40 587–590. 10.1130/G32923.1

[B24] Land L. S. (1998). Failure to precipitate dolomite at 25°C from dilute solution despite 1000-fold oversaturation after 32 years. *Aquat. Geochem.* 4 361–368. 10.1023/A:1009688315854

[B25] Liu H. Buskey E. J. (2000). Hypersalinity enhances the production of extracellular polymeric substance (eps) in the texas brown tide alga, Aureoumbra Lagunensis (PELAGOPHYCEAE). *J. Phycol.* 36 71–77. 10.1046/j.1529-8817.2000.99076.x

[B26] McGenity T. J. Gemmell R. T. Grant W. D. (1998). Proposal of a new halobacterial genus Natrinema gen. nov., with two species Natrinema pellirubrum nom. nov. and Natrinema pallidum nom. nov. *Int. J. Systemat. Evol. Microbiol.* 48 1187–1196. 10.1099/00207713-48-4-1187 9828420

[B27] McKenzie J. A. (1981). Holocene dolomitization of calcium carbonate sediments from the coastal sabkhas of Abu Dhabi, UAE: a stable isotope study. *J. Geol.* 89 185–198. 10.1086/628579

[B28] McKenzie J. A. Vasconcelos C. (2009). Dolomite mountains and the origin of the dolomite rock of which they mainly consist: historical developments and new perspectives. *Sedimentology* 56 205–219. 10.1111/j.1365-3091.2008.01027.x

[B29] Meister P. Reyes C. Beaumont W. Rincon M. Collins L. Berelson W. (2011). Calcium and magnesium-limited dolomite precipitation at Deep Springs Lake, California. *Sedimentology* 58 1810–1830. 10.1111/j.1365-3091.2011.01240.x

[B30] Mitchell J. Land L. S. Miser D. E. (1987). Modern marine dolomite cement in a north Jamaican fringing reef. *Geology* 15 557–560. 10.1130/0091-7613(1987)15<557:MMDCIA>2.0.CO;2

[B31] Morrow D. (1978). The Influence of the Mg/Ca Ratio and salinity on dolomitization in evaporite basins: geological note. *Bull. Can. Pet. Geol.* 26 389–392.

[B32] Morrow D. W. Ricketts B. D. (1988). “Experimental investigation of sulfate inhibition of dolomite and its mineral analogues,” in *Sedimentology and Geochemistry of Dolostones* eds Shukla V. Baker P. A. (Tulsa: SEPM Special Publication) 27–38. 10.2110/pec.88.43.0025

[B33] Nutman A. P. Friend C. R. L. Bennett V. C. Wright D. Norman M. D. (2010). > = 3700 Ma pre-metamorphic dolomite formed by microbial mediation in the Isua supracrustal belt (W. Greenland): simple evidence for early life? *Precambrian Res.* 183 725–737. 10.1016/j.precamres.2010.08.006

[B34] Oren A. (2013). “Life at high salt concentrations,” in *The Prokaryotes–Prokaryotic Communities and Ecophysiology* eds Rosenberg E. DeLong E. Lory S. Stackebrandt E. Thompson F. (Berlin: Springer) 421–440.

[B35] Peterson M. Bien G. Berner R. (1963). Radiocarbon studies of recent dolomite from Deep Spring Lake, California. *J. Geophys. Res.* 68 6493–6505. 10.1029/JZ068i024p06493

[B36] Pina C. M. (2015). Reaction pathways toward the formation of dolomite. *Am. Mineral.* 100 1017–1018. 10.2138/am-2015-5269

[B37] Qiu X. Wang H. Liu D. Gong L. Wu X. Xiang X. (2012). The physiological response of Synechococcus elongatus to salinity: a potential biomarker for ancient salinity in evaporative environments. *Geomicrobiol. J.* 29 477–483. 10.1080/01490451.2011.581331

[B38] Qiu X. Wang H. Yao Y. Duan Y. (2017). High salinity facilitates dolomite precipitation mediated by Haloferax volcanii DS52. *Earth Planet. Sci. Lett.* 472 197–205. 10.1016/j.epsl.2017.05.018

[B39] Rivadeneyra A. Rivadeneyra M. A. Escamilla C. V. Algarra A. M. Navas A. S. Martín-Ramos J. D. (2016). The influence of salt concentration on the precipitation of magnesium calcite and calcium dolomite by Halomonas anticariensis. *Expert Opin. Environ. Biol.* 4 1–9. 10.4172/2325-9655.1000130

[B40] Roberts J. A. Bennett P. C. Gonzalez L. A. Macpherson G. L. Milliken K. L. (2004). Microbial precipitation of dolomite in methanogenic groundwater. *Geology* 32 277–280. 10.1130/G20246.2

[B41] Rodriguez-Blanco J. D. Shaw S. Benning L. G. (2015). A route for the direct crystallization of dolomite. *Am. Mineral.* 100 1172–1181. 10.2138/am-2015-4963

[B42] Rosen M. R. Miser D. E. Starcher M. A. Warren J. K. (1989). Formation of dolomite in the Coorong region, South Australia. *Geochim. Cosmochim. Acta* 53 661–669. 10.1016/0016-7037(89)90009-4

[B43] Sánchez-Román M. McKenzie J. A. de Luca Rebello Wagener A. Rivadeneyra M. A. Vasconcelos C. (2009). Presence of sulfate does not inhibit low-temperature dolomite precipitation. *Earth Planet. Sci. Lett.* 285 131–139. 10.1016/j.epsl.2009.06.003

[B44] Shen P. Chen Y. (1994). Plasmid from Halobacterium halobium and its restrictionmap. *Yi Chuan Xue Bao* 21 409–416. 7848666

[B45] Smith R. M. Martell A. E. (1976). “Inorganic complexes,” in *Critical Stability Constants* eds Smith R. M. Martell A. E. (New York, NY: Plenum Press) 1–257. 10.1007/978-1-4757-5506-0

[B46] Tourney J. Ngwenya B. T. (2015). The role of bacterial extracellular polymeric substances in geomicrobiology. *Chem. Geol.* 386 115–132. 10.1128/AEM.06568-11 22179248 PMC3273019

[B47] Van Lith Y. Vasconcelos C. Warthmann R. Martins J. C. F. McKenzie J. A. (2002). Bacterial sulfate reduction and salinity: two controls on dolomite precipitation in Lagoa Vermelha and Brejo do Espinho (Brazil). *Hydrobiologia* 485 35–49. 10.1023/a:1021323425591

[B48] Van Tuyl F. M. (1916). The present status of the dolomite problem. *Science* 44 688–690. 10.1126/science.44.1141.688 17780053

[B49] Vasconcelos C. McKenzie J. A. (1997). Microbial mediation of modern dolomite precipitation and diagenesis under anoxic conditions (Lagoa Vermelha, Rio de Janeiro, Brazil). *J. Sediment. Res.* 67 378–390.

[B50] Vasconcelos C. McKenzie J. A. Bernasconi S. Grujic D. Tien A. J. (1995). Microbial mediation as a possible mechanism for natural dolomite formation at low temperatures. *Nature* 377 220–222. 10.1038/377220a0

[B51] Voegerl R. S. (2014). *Quantifying the Carboxyl Group Density of Microbial Cell Surfaces as a Function of Salinity: Insights into Microbial Precipitation of Low-Temperature Dolomite*. Ph.D. thesis, University of Kansas Lawrence.

[B52] Wang X. Chou I. M. Hu W. Yuan S. Liu H. Wan Y. (2016). Kinetic inhibition of dolomite precipitation: insights from Raman spectroscopy of Mg2+–SO42- ion pairing in MgSO4/MgCl2/ NaCl solutions at temperatures of 25 to 200°C. *Chem. Geol.* 435 10–21. 10.1016/j.chemgeo.2016.04.020

[B53] Warren J. (2000). Dolomite: occurrence, evolution and economically important associations. *Earth Sci. Rev.* 52 1–81. 10.1016/S0012-8252(00)00022-2

[B54] Warthmann R. van Lith Y. Vasconcelos C. McKenzie J. A. Karpoff A. M. (2000). Bacterially induced dolomite precipitation in anoxic culture experiments. *Geology* 28 1091–1094. 10.1130/0091-7613(2000)28<1091:BIDPIA>2.0.CO;2

[B55] Yao Y. C. Qiu X. Duan Y. Wang H. M. (2018). Dolomite formation mediated by halophilic archaeal cells under different conditions and carboxylated microspheres. *Earth Sci.* 43 449–458. 10.3799/dqkx.2017.579

[B56] Zengler D. Dunham J. Ethington R. L. (1980). *Concepts and Models of Dolomitization. The Chemistry of Dolomite Formation I: the Stability of Dolomite, Society of Economic Paleontologists and Mineralogists* Vol. 28. London: Special Publications 111–121. 10.2110/pec.80.28

[B57] Zhang F. Xu H. F. Konishi H. Roden E. E. (2010). A relationship between d104 value and composition in the calcite-disordered dolomite solid-solution series. *Am. Mineral.* 95 1650–1656. 10.2138/am.2010.3414

[B58] Zhang F. F. Xu H. F. Konishi H. Shelobolina E. S. Roden E. E. (2012). Polysaccharide-catalyzed nucleation and growth of disordered dolomite: a potential precursor of sedimentary dolomite. *Am. Mineral.* 97 556–567. 10.2138/am.2012.3979

